# Comprehensive Health Risk Assessment of Electronic Cigarette Aerosols: Metal and PAH Characterization, Oxidative Potential, and Cancer Risk Estimation

**DOI:** 10.1021/acsenvironau.5c00304

**Published:** 2026-04-15

**Authors:** Li-Ti Chou, Tsai-Ling Chen, Jen-Kun Chen, Hsiao-Chi Chuang, Kai-Chien Yang, Ta-Chih Hsiao

**Affiliations:** † Graduate Institute of Environmental Engineering, 33561 National Taiwan University, Taipei 106319, Taiwan; ‡ Institute of Biomedical Engineering and Nanomedicine, National Health Research Institutes, Miaoli 350401, Taiwan; § School of Respiratory Therapy, College of Medicine, Taipei Medical University, Taipei 110301, Taiwan; ∥ National Heart and Lung Institute, Imperial College London, London SW3 6LY, U.K.; ⊥ Division of Pulmonary Medicine, Department of Internal Medicine, Shuang Ho Hospital, Taipei Medical University, New Taipei City 235041, Taiwan; # Cell Physiology and Molecular Image Research Center, Wan Fang Hospital, Taipei Medical University, Taipei 110301, Taiwan; ∇ Department and Graduate Institute of Pharmacology, National Taiwan University College of Medicine, Taipei 106319, Taiwan; ○ Research Center for Developmental Biology & Regenerative Medicine, National Taiwan University, Taipei 106319, Taiwan; ◆ Division of Cardiology, Department of Internal Medicine and Cardiovascular Center, National Taiwan University Hospital, Taipei 100225, Taiwan; ¶ Institute of Biomedical Sciences, Academia Sinica, Taipei 115201, Taiwan; & Research Centre for Environmental Changes, Academia Sinica, Taipei 115201, Taiwan; ● Institute of Atomic and Molecular Sciences, Academia Sinica, Taipei 115201, Taiwan

**Keywords:** electronic cigarettes (ECs), particle size distribution (PSD), polycyclic aromatic hydrocarbons (PAHs), inhalation risk assessment, oxidative potential (OP), excess lifetime cancer risk (ELCR)

## Abstract

Electronic cigarettes (ECs) have emerged as popular alternatives to traditional cigarettes (TCs), yet their health impacts remain contentious due to limited systematic evaluations integrating multiple toxicological end points. This study provides a comprehensive assessment of EC aerosol toxicity by combining chemical characterization, oxidative potential (OP) measurement, and health risk modeling with systematic cross-study comparisons. Chemical analysis revealed that five metals (Cu, Ni, Ag, Zn, and Pb) originate exclusively from device components, while cross-study comparison identified substantial heterogeneity in metal profiles across different devices and studies. PAH analysis detected 14 compounds with a total concentration of 1.373 ng/puff. OP measurements demonstrated a marked dichotomy between OPv (49.87 nmol/min/m^3^) and OPm (0.02 pmol/min/μg), indicating that particle number and surface area, rather than mass alone, are key determinants of EC aerosol oxidative toxicity. ELCR estimates ranged across 3 orders of magnitude (10^–6^ to 10^–3^), with chromium oxidation state (Cr^3+^ vs Cr^6+^) representing the single largest source of uncertainty. Compared to TC emissions, which are primarily driven by combustion, EC aerosols represent a fundamentally different type of exposure whose chemical composition and health risks vary greatly with device design, coil materials, and operating conditions. These findings demonstrate that EC risk is device-structured and endpoint-specific, and that risk characterization depends critically on the choice of chemical species, analytical panels, and exposure metrics being evaluated. Therefore, comprehensive and rigorous evaluation of EC health risks requires careful consideration.

## Graphical abstract

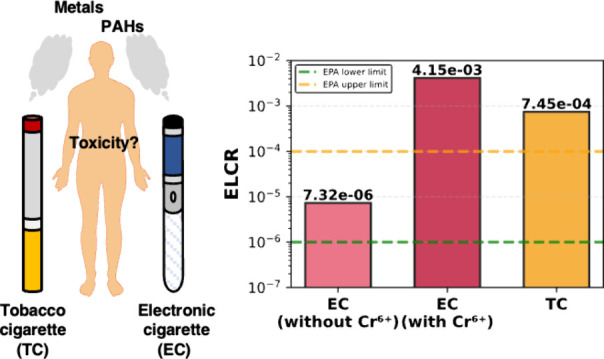

## 1 Introduction

Electronic cigarettes (ECs) have rapidly gained popularity as an alternative to traditional cigarettes (TCs), primarily due to their perceived safety and reduced harmful emissions.^1–3^ Unlike TCs, which rely on combustion, ECs operate by heating an e-liquid, typically composed of humectants (propylene glycol and vegetable glycerin), nicotine, and flavorings, using a battery-powered coil.^4^ This process, often marketed as a less harmful means of nicotine consumption, has led to widespread adoption among both former smokers and new users, including adolescents.^5^ Although ECs operate via a noncombustion mechanism, they still produce complex aerosols containing potentially toxic substances, including metals and PAHs, which pose health risks.^6–8^ While initial studies suggested ECs might pose fewer risks than TCs, emerging evidence indicates that their aerosol composition and particle size distribution (PSD) may contribute to respiratory and cardiovascular issues.^9–12^ In particular, the inhalation of fine and ultrafine particles raises concerns about potential toxicological effects, as these particles may contain hazardous metals such as chromium, nickel, and lead leached from device components, as well as organic compounds including formaldehyde, acrolein, and benzene generated from e-liquid thermal degradation.^13–15^ Therefore, a comprehensive investigation into the physical and chemical properties of EC aerosols is essential to accurately assess their impact on human health and compare their risks with those of TCs.

Existing research on EC aerosols has primarily focused on characterizing their chemical constituents and evaluating their potential toxicological effects. Several studies have identified metals such as chromium (Cr), nickel (Ni), and lead (Pb) in EC aerosols, originating from heating elements and other device components.^7,16^ These metals can enter the respiratory tract upon inhalation, posing potential health risks, particularly for chronic users.^17,18^ Additionally, the thermal degradation of e-liquid components, including vegetable glycerin, propylene glycol, and flavoring agents, can generate reactive oxygen species (ROS, a group of highly reactive oxygen-derived molecules known to cause oxidative damage^19^) and other toxic byproducts, such as aldehydes and volatile organic compounds (VOCs).^20–22^ The presence of polycyclic aromatic hydrocarbons (PAHs) in EC aerosols, although generally lower than in TCs, remains a significant concern due to their established carcinogenicity.^23–25^ Moreover, the PSD of EC aerosols plays a critical role in determining their deposition in the human respiratory system.^26,27^ Studies indicate that EC aerosols exhibit hygroscopic properties, meaning their particle size can change in humid environments, such as the lungs.^12,28^ This characteristic influences the extent and location of particle deposition, affecting the overall toxicity profile of EC aerosols. Furthermore, oxidative stress induced by EC aerosols has been implicated in inflammation and endothelial dysfunction, factors closely associated with cardiovascular diseases.^29,30^ Despite these insights, current research faces challenges in standardizing methodologies for EC aerosol analysis, particularly regarding variations in device configurations, e-liquid compositions, and user behaviors. As a result, there remains a critical need for a systematic approach to evaluate the chemical profiles of EC aerosols, their inhalation dosimetry, and their long-term health implications.

This study aims to provide a comprehensive assessment of health risks associated with EC aerosols. We first characterize the chemical composition of EC aerosols, focusing on metals and PAHs. PSDs are then measured and incorporated into the multiple-path particle dosimetry (MPPD) model to estimate size-resolved deposition in the human respiratory tract. Based on deposition-adjusted doses, excess lifetime cancer risk (ELCR) is calculated to evaluate the carcinogenic potential of EC emissions relative to TCs. Additionally, oxidative potential (OP) is assessed to further characterize the capacity of EC aerosols to induce oxidative stress.

## 2 Materials and Methods

### 2.1 EC Aerosol Generation and Multiple-Path Particle Dosimetry Model (MPPD)

A tank-style EC system was utilized as illustrated in Figure S1, following the experimental setup detailed in ref 27. The EC system was equipped with a 0.6-ohm mesh coil heater and relied on a custom power supply for precise puff regulation. Locally available, nicotine-free e-liquid cartridges containing 60.6% vegetable glycerin, 20.2% propylene glycol, and 19.2% flavor additives were used. The puff protocol consisted of 5 V operating voltage, 2-s puff duration, 1 min interpuff interval, and 40 mL puff volume, generating 240 puffs per day. The 1 min interpuff interval was implemented to allow sufficient coil cooling between puffs and prevent overheating. Additionally, a 15 min break was provided every hour, during which the e-liquid was refilled. The heating coil was replaced every 3 days to prevent coil degradation. For homogeneous mixing of puffs, air was introduced at a flow rate of 8 L/min before entering the exposure chamber. System maintenance included refilling the e-liquid and replacing the coil every 3 days to prevent coil burnout. Aerosol particle size distributions were measured using two Scanning Mobility Particle Sizers (SMPS, Model 3082, TSI, USA) at designated flow rates to ensure accurate characterization. Inhalation doses in both mouse (B6C3F1) and human respiratory systems were determined using the Multiple-Path Particle Dosimetry (MPPD) model. For human evaluations, the Yeh/Schum-5-Lobe model was applied as referenced in Asgharian, Hofmann,^31^ and standard parameters were retained for other lung attributes.

### 2.2 Chemical Composition Analysis

#### 2.2.1 Metal Composition Analysis

For each test, 20 puffs of EC aerosols were sampled onto a PTFE filter. This procedure was repeated five times to obtain five independent filter samples. After collection, each filter was stored in a glass Petri dish at −20 °C until analysis. Filter weighing was performed at least twice before and after sampling using a microbalance under controlled conditions (25 ± 2 °C, relative humidity 40 ± 5%) following a 24-h equilibration period. Seven blank samples were prepared by capturing HEPA-filtered clean air for 20 min at a flow rate of 8 L/min. Each collected sample underwent digestion with 20 mL of 69.0–70.0% HNO_3_ in an Advanced Microwave Digestion System (ETHOS EASY, Milestone, Italy) at 160 °C for 30 min. Subsequently, Inductively Coupled Plasma-Mass Spectrometry (ICP-MS, NexION 2000, PerkinElmer, USA) was employed to determine the concentrations of 19 elements following the Taiwan National Environmental Research Academy standardized method (NIEA A305.12C). Calibration was performed using serial dilutions of high-purity multielement standards (23 elements, 1000 μg/mL in 1 mol/L HNO_3_, AccuStandard). For V, a separate standard (1000 μg/mL in 2–5% HNO_3_, AccuStandard) was used. The method detection limit (MDL) was defined as three times the standard deviation of the blank samples. The 19 target elements and their corresponding MDLs for both aerosol and e-liquid samples are summarized in Table S1. For the analysis of metal components in e-liquids, an identical analytical procedure was performed with three replicate measurements. Seven blank samples prepared with deionized (DI) water were used for background correction.

#### 2.2.2 PAH Composition Analysis

For PAH analysis, 60 puffs of EC aerosols were captured using quartz filters previously baked at 400 °C for 16 h to eliminate organic impurities. Sample storage, weighing, and blank preparation followed the same procedures described in Section 2.2.1, except that blank samples were collected for 60 min. The extraction process for 26 PAH types utilized the Soxhlet method with a hexane to acetone ratio of 1:1 for 8 h. PAH analysis was conducted using Gas Chromatography-tandem Mass Spectrometry (GC–MS/MS, TRACE 1310, TSQ 8000 Evo, Thermo, USA) following the Taiwan National Environmental Research Academy standardized method (NIEA A801.90C). This experimental procedure was executed three times for consistency.

### 2.3 Toxic Equivalency Factors (TEFs)

To assess the toxicity levels of PAHs, toxic equivalency factors (TEFs) were employed to evaluate the potential carcinogenicity of selected PAHs. Each PAH’s TEF indicates its potency relative to benzo­[a]­pyrene, which has a unity TEF as established by Nisbet and Lagoy.^32^ The detection limits and TEFs for all analyzed PAH types are provided in Table S2. The toxic equivalent quantity (TEQ), denoted as BaPeq, was computed by accumulating the products of each PAH concentration (PAH_*i*_) and its associated TEF (TEF_*i*_), following eq 1:

$$\mathrm{BaPeq}=\Sigma {\mathrm{PAH}}_{i}\times {\mathrm{TEF}}_{i}$$ 1

### 2.4 Oxidative Potential (OP) Measurement

The dithiothreitol (DTT) assay quantifies aerosol OP by measuring the capacity of particulate matter to generate reactive oxygen species (ROS) in vitro. This method was validated using 9,10-phenanthraquinone (PQN) as a positive control following the protocols of^33^ with modification before application to EC aerosols. EC aerosols and HEPA-filtered controls were collected on quartz filters for 1 h, extracted in 15 mL DI water via 30 min sonication, and filtered through 0.45 μm membranes to remove insoluble particles. The assay was conducted in five replicate tubes, each containing 0.5 mL of sample extract, 1 mL of potassium phosphate buffer (0.1 M, pH 7.4), and 125 μL of DTT solution (0.5 mM). Tubes were incubated at 37 °C with agitation at 200 rpm. At 8 min intervals, one tube was removed and immediately mixed with 0.5 mL 5,5′-dithiobis­(2-nitrobenzoic acid) (DTNB) solution (0.5 mM), which reacts with residual DTT to form TNB. The absorbance of 2-nitro-5-thiobenzoic acid (TNB) was measured at 412 nm using UV–vis spectrophotometry (Evolution 201, Thermo, USA). The five absorbance measurements over time yielded a linear decrease, from which the DTT consumption rate (σDTT, nmol/min) was calculated via linear regression according to eq 2:

$$\mathrm{\sigma DTT}=-\mathrm{\sigma Abs}\cdot \frac{N_{0}}{{\mathrm{Abs}}_{0}}$$ 2

where σAbs is the absorbance–time slope, Abs_0_ is the initial absorbance, and *N*_0_ (nmol) represents the initial moles of DTT.

OP activity was determined by blank subtraction and normalization to either sampling volume or particle mass, as shown in eq 3:

$$\mathrm{OP}=\frac{{\mathrm{\sigma DTT}}_{\mathrm{sample}}-{\mathrm{\sigma DTT}}_{\mathrm{blank}}}{V_{\mathrm{air}}(\mathrm{or}\;M_{\mathrm{particle}})}$$ 3

where σDTT_sample_ is the DTT consumption rate of the aerosol sample (nmol/min), σDTT_blank_ is the DTT consumption rate of the blank sample (nmol/min), *V*_air_ is the sampling volume (m^3^) for volume-normalized OP (OPv, nmol/min/m^3^), and *M*_particle_ is the total particle mass (μg) for mass-normalized OP (OPm, pmol/min/μg).

### 2.5 Excess Lifetime Cancer Risk (ELCR)

The ELCR quantifies the additional probability that an individual will develop cancer due to prolonged exposure to carcinogens over a typical lifespan of 70 years. Given the heightened risks associated with ultrafine particles (UFPs), a nuanced risk model proposed by Sze-To, Wu^34^ was adopted. This model prioritizes particle surface area as the dosimetry metric for UFPs and introduces a conversion coefficient (*C*_f_) to bridge the relationship between surface area-based and conventional mass-based cancer potency. The ELCR was calculated according to eq 4:

$$\mathrm{ELCR}=\frac{\Sigma ({\mathrm{SF}}_{i}\times m_{i})}{\mathrm{BW}\times \mathrm{PM}}\times (C_{f}\times \delta_{\mathrm{SA}}+\delta_{\mathrm{PM}})\times V_{\mathrm{inhaled}}\times \frac{Y}{70}$$ 4

where SF_*i*_ (mg/kg-day)^−1^ is the slope factor describing the mass-based cancer potency of the *i*-th pollutant obtained from OEHHA^35^ (Table S3), *m_i_* (mg/m^3^) is the mass concentration of the *i*-th pollutant, BW (kg) is the body weight (assumed 60 kg for adults), PM (mg/m^3^) is the particle mass concentration, *C*_f_ (mg/nm^2^) is the conversion coefficient (6.60 × 10^–13^ mg/nm^2^) obtained from Sze-To, Wu,^34^ δ_SA_ (nm^2^/m^3^) is the total surface area of particles less than 100 nm deposited in the tracheobronchial and pulmonary regions, δ_PM_ (mg/m^3^) is the total mass of particles greater than 100 nm deposited in the tracheobronchial and pulmonary regions, *V*_inhaled_ (m^3^/day) is the inhaled puff volume per day (8.68 × 10^–3^ m^3^/day for EC users), and *Y* represents the smoking duration in years (24.3 years based on Stabile, Buonanno^36^). Detailed exposure conditions and parameters are provided in Table 1.

**Table 1:** EC Use Characteristics^37^ ^,^ ^a^

participant characteristic (*n* = 14)	mean (or %)	EC use characteristics	mean [95% CI]
age (years)	19.7	puff duration (s)	3.3 [2.3–4.3]
male	92.9%	puff interval (s)	38.1 [24.7–51.4]
white race	64.3%	puff volume (mL)	110.3 [10.4–150.3]
		puff flow rate (mL/s)	26.6 [23.1–30.1]
		total daily puffs	76.6 [38.7–114.4]
		total daily puff volume (mL)	8682.5 [2709.2–14,655.8]

a Note: Only reported the EC use characteristics of established cigarette smokers on weekdays. Subjects who reported smoking “at least 100 tobacco cigarettes” in their lifetime were categorized as established smokers.

## 3 Results and Discussion

### 3.1 EC Aerosol Chemical Compositions

#### 3.1.1 Metals

A detailed examination of 19 metals in e-liquid and aerosols revealed distinct distribution patterns. Metal concentrations in e-liquid and aerosols are presented in Figure 1, with detailed values provided in Table S4. Seven metals (As, Cd, Cs, Co, Ga, In, Tl) consistently registered below detectable limits in both matrices. The remaining 12 metals exhibited two distinct distribution patterns: seven metals (Al, Ba, Cr, Mn, Se, V, Fe) were detected in both e-liquids and aerosols, while five metals (Cu, Ni, Ag, Zn, Pb) were detected exclusively in aerosols. The detection of Al, Ba, Cr, Mn, Se, V, and Fe in both matrices suggests that e-liquid ingredients serve as the primary source for these metals, likely originating from tobacco flavoring derived from plants that naturally absorb metals from soil.^38^ Among these, Cr warrants particular attention. Ranking first in e-liquids and third in aerosols, Cr raises concerns about potential oxidative conversion from Cr^3+^ to carcinogenic Cr^6+^ (IARC Group 1) in the pulmonary environment.^39,40^

**Figure 1:**
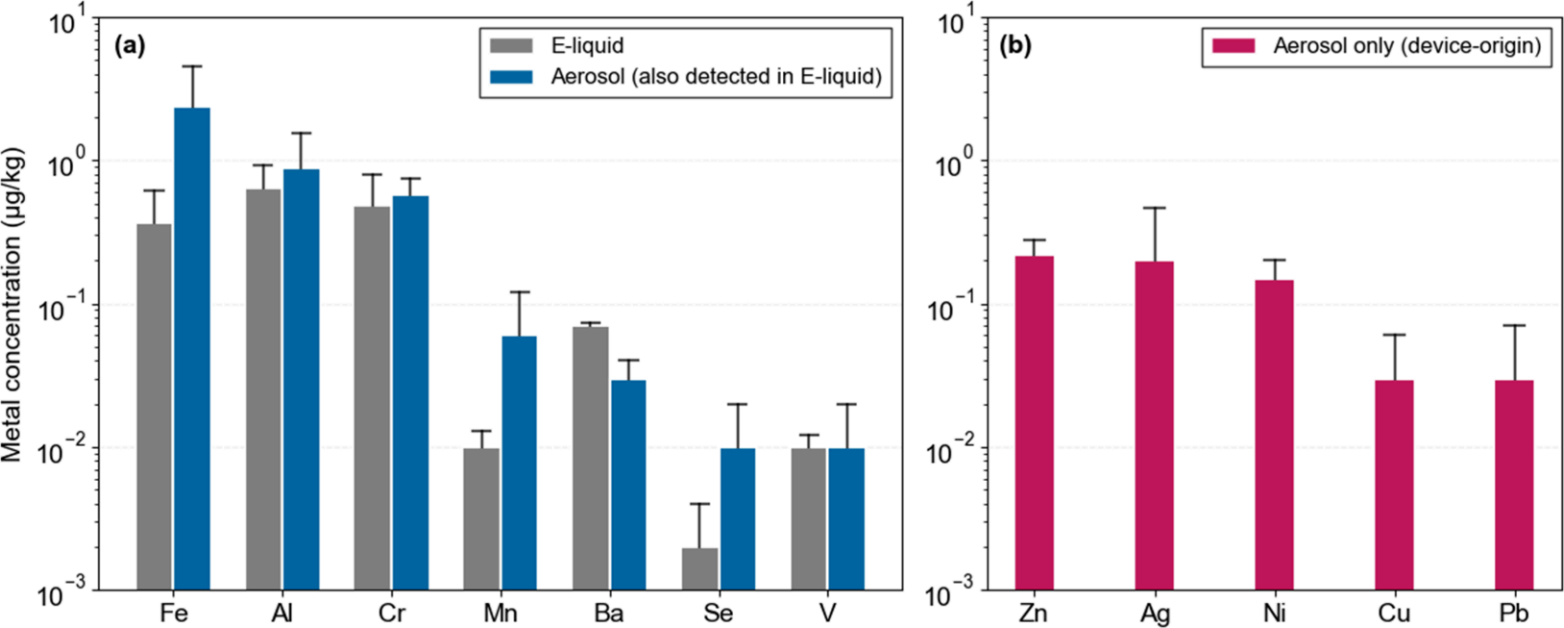
Metal concentrations (μg/kg) in e-liquid and aerosol from electronic cigarettes. (a) Metals detected in both e-liquid and aerosol; gray and blue bars represent e-liquid and aerosol concentrations, respectively. (b) Metals detected exclusively in aerosol, indicating device-origin contamination likely derived from heating coil or atomizer components. Error bars represent one standard deviation.

In contrast, the exclusive presence of Cu, Ni, Ag, Zn, and Pb in aerosols strongly implicates device components as the contamination source. To test this hypothesis, we compared our findings with Williams, Bozhilov,^6^ who characterized the elemental composition of various EC device components using energy dispersive X-ray spectrometry (EDS), including the Smok tank used in the present study (Table 2). Their analysis identified Cu, Ni, and Zn in structural components such as filaments, wires, and mesh rings. The concordance between metals present in device hardware and those detected exclusively in our aerosol samples supports the hypothesis that these metals are released directly from device surfaces during heating. This finding carries significant health implications, particularly for Ni given its IARC Group 2B classification.^41,42^ Ag and Pb, though not reported by Williams, Bozhilov,^6^ were also detected exclusively in our aerosol samples. The presence of Ag likely reflects device-specific materials, as Ag is commonly applied as a coating on heating coils to enhance electrical conductivity in certain EC models. For Pb, although Pb-containing solder is now prohibited in Chinese manufacturing,^7,43^ its exclusive detection in aerosols suggests that Pb may originate from device components and is released through thermal cycling during repeated heating and cooling. It should be noted that some metals (Al, Cr, Mn, Fe) were detected in device components by Williams, Bozhilov^6^ yet also present in our e-liquid samples. For these metals, both e-liquid ingredients and device leaching may contribute to aerosol concentrations, and the relative contribution of each source cannot be unambiguously resolved. Based on these findings, we propose three metal contamination pathways. First, metals naturally present in e-liquid ingredients become aerosolized during heating. Second, device components in prolonged contact with e-liquid may leach metals, which are subsequently aerosolized. Third, thermal cycling from repeated heating and cooling promotes direct release of metal particles from device surfaces.

**Table 2:** Metal Elements in EC Device Components^a^

Component	Elements	Component	Elements
filament	Ni, Cr, Fe	air-tube	Ni, Fe, Zn, Sn, Co, Cu,
thick wire	Ni	wick	Al, Si, O, Ca, Na, Ti
wire-to-wire joint	Ni, Cr, Al, Si, O (minor: Mn, Ti)	mesh ring	Fe, Cr, Ni, Mn, Cu

a The table shows elemental composition detected in different structural components of electronic cigarette devices using energy dispersive X-ray spectrometry (EDS).

Comparison with three previous studies^17,18,44^ revealed both consistent and divergent metal profiles (Figure 2 and Table S5). Al, Cr, Ni, Zn, Pb, and Fe were consistently detected across studies, suggesting that these metals represent common contaminants in EC aerosols regardless of device type. In contrast, Ba, Se, Ag, and V were detected only in the present study, which may reflect differences in device materials, e-liquid formulations, or analytical sensitivity across investigations. Recent analyses of modern disposable devices have documented substantial increases in Pb, Ni, Cr, and Sb concentrations with prolonged device use, with certain brands exceeding established screening thresholds.^45,46^ These findings underscore the critical role of device materials and usage duration in determining metal exposure profiles.

**Figure 2:**
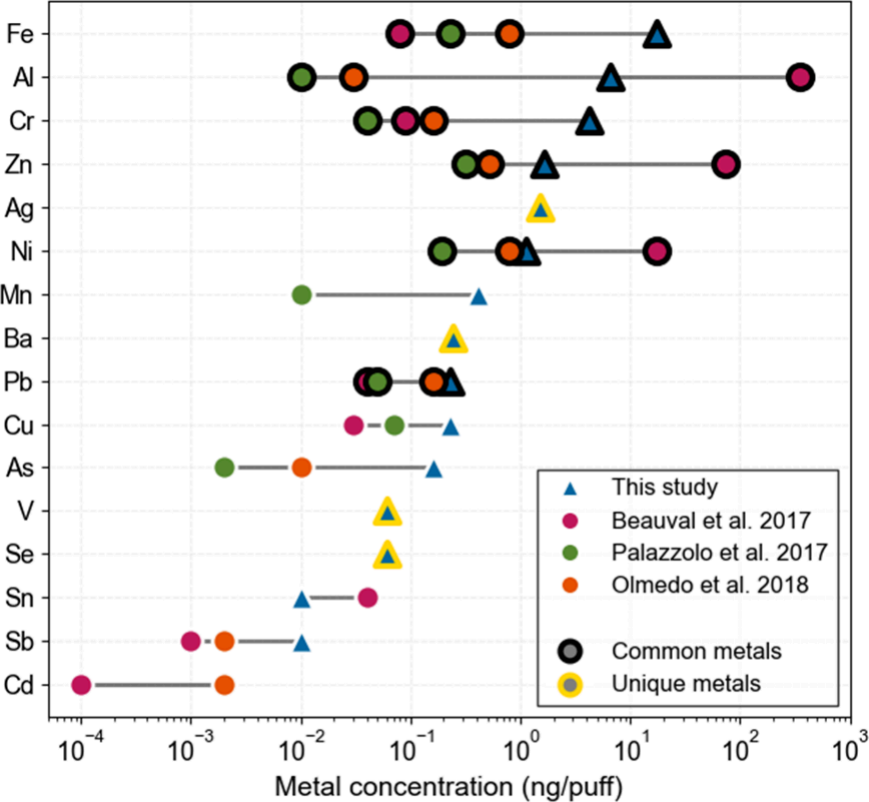
Metal concentrations (ng/puff) in EC aerosols across studies. Common metals (black borders) were detected in multiple studies, while unique metals (gold borders) were specific to this study.

The comparison between EC and TC reveals fundamentally different metal origins (Figure 3 and Table S6). EC metals predominantly originate from device components, whereas TC metals largely derive from tobacco plants.^47^ Despite these different sources, both delivery systems expose users to potentially harmful metals. To assess the toxicological relevance of these metal exposures, we performed a risk assessment using Minimal Risk Levels (MRLs) established by the Agency for Toxic Substances and Disease Registry (ATSDR) for inhalation exposure.^48^ The measured metal concentrations were adjusted by multiplying with human respiratory tract deposition fractions determined in our previous study using the MPPD model: 15.44% for TC smoke and 18.60% for EC aerosols.^27^ After deposition adjustment, distinct patterns of MRL exceedance emerged (Table 3). For EC aerosols, Cr, Mn, Ni, and V exceeded their respective MRLs, with Cr showing the largest exceedance at approximately 195-fold above the threshold. For TC aerosols, Cd, Mn, and Ni exceeded MRLs, with Cd and Mn exhibiting exceedances of over 1000-fold. Notably, Pb lacks a defined MRL due to its toxicity at any exposure level.^49^ These findings demonstrate that both EC and TC aerosols pose metal-related health risks, though the specific metals of concern differ between the two systems. These results underscore that metal exposure is a critical consideration in evaluating the health risks of EC use.

**Table 3:** Metal Concentrations in EC and TC Aerosols Relative to ATSDR Inhalation MRLs^a^

metal	ATSDRMRL of inhalation (mg/m^3^)	EC (mg/m^3^)	TC (mg/m^3^)
Cr^b^	1 × 10^–4^	1.95 × 10^–2^	
Cd^c^	1 × 10^–5^	<LOD	1.74 × 10^–2^
Co	1 × 10^–4^	<LOD	3.80 × 10^–5^
Mn	3 × 10^–4^	1.89 × 10^–3^	3.07 × 10^–1^
Ni^d^	9 × 10^–5^	5.21 × 10^–3^	2.51 × 10^–4^
V^c^	1 × 10^–4^	2.92 × 10^–4^	

a Metal concentrations (mg/m^3^) are compared against inhalation MRLs to assess potential health risks. Values below the limit of detection are indicated as <LOD.

b MRL of Cr^3+^ soluble particulates.

c MRL of acute inhalation.

d MRL of chronic inhalation.

**Figure 3:**
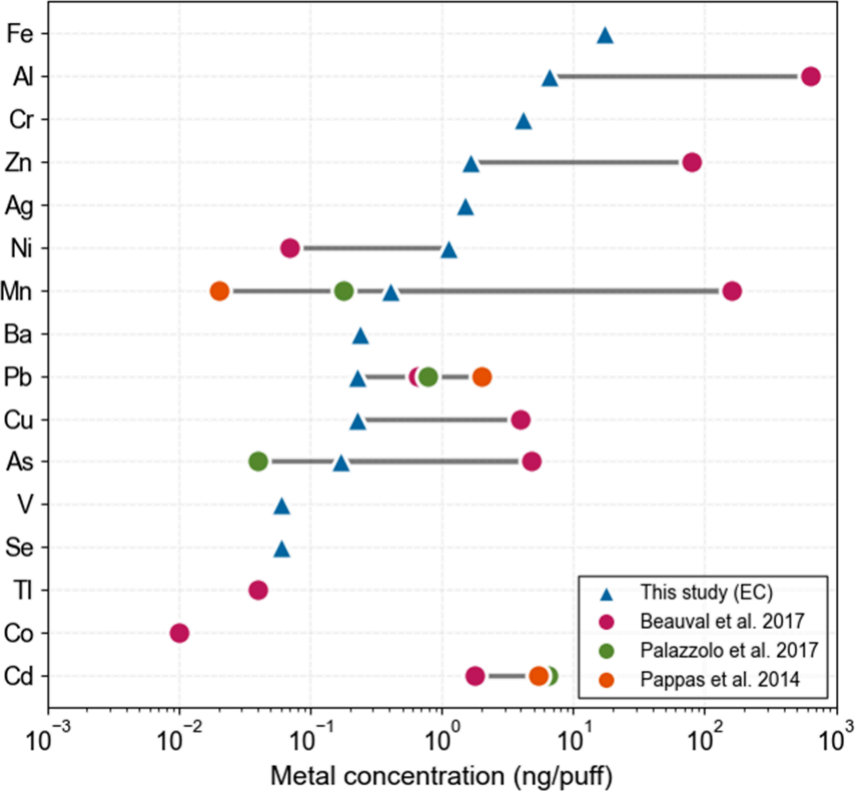
Metal concentrations (ng/puff) in EC versus TC aerosols across studies.

#### 3.1.2 Polycyclic Aromatic Hydrocarbons (PAHs)

Our comprehensive analysis covered 26 distinct PAH types, of which 14 were detected in EC aerosols. Concentrations are expressed as ng/puff with a puff volume of 40 mL (Table 4). To evaluate the carcinogenic potential of EC aerosols, we first examined the seven PAHs classified as Group B2 probable human carcinogens by the U.S. EPA, including benz­[a]­anthracene, benzo­[b]­fluoranthene, benzo­[k]­fluoranthene, benzo­[a]­pyrene, chrysene, dibenz­[a,h]­anthracene, and indeno­[1,2,3-cd]­pyrene. Among these, three were detected in our EC aerosol samples, specifically benzo­[b]­fluoranthene (0.000 ± 0.004 ng/puff), benzo­[a]­pyrene (0.015 ± 0.018 ng/puff), and dibenz­[a,h]­anthracene (0.003 ± 0.002 ng/puff), while benz­[a]­anthracene, benzo­[k]­fluoranthene, chrysene, and indeno­[1,2,3-cd]­pyrene remained below the detection limit. Comparison with previous studies reveals that all seven B2 PAHs are consistently detected at substantially higher concentrations in TC aerosols than in EC aerosols (Table 4), reflecting the fundamental difference between combustion-driven PAH formation in TCs (>800 °C^50^) and thermal vaporization in ECs (∼300 °C^51^).

**Table 4:** Comparison of PAH Concentrations (ng/puff) in EC and TC Aerosols across Studies^a^ ^,^ ^b^

	this study	Dusautoira, Zarconea^23^		Rankin, Wingfors^24^		Margham, McAdam^14^		Tayyarah and Long^53^	
PAHs (ng/puff)	EC	EC	TC	EC	TC	EC	TC	EC	TC
>LOD types ratio	14/26	20/21	20/21	5/12	12/12	2/14	14/14	3/15	15/15
naphthalene	<LOD	0.056	2.617			0.020	70.961	0.238	91.896
2-methylnaphthalene	<LOD								
acenaphthylene	<LOD							<LOD	14.618
acenaphthene	0.017 ± 0.013	0.002	0.959					0.104	8.339
fluorene	0.150 ± 0.078	0.004	1.438					<LOD	23.256
phenanthrene	<LOD	0.013	2.058	0.142	26.182			0.032	16.655
anthracene	0.061 ± 0.027	0.001	0.986	<LOD	17.455			<LOD	7.709
fluoranthene	<LOD	0.010	1.064	0.066	21.818			<LOD	10.133
pyrene	0.000 ± 0.001	0.019	1.274	0.095	15.273			<LOD	9.387
benzo[c]fluorene	0.003 ± 0.005	0.002	0.001						
*benz[a]anthracene	<LOD	0.001	0.395	0.088	8.000	<LOD	1.770	<LOD	2.473
cyclopenta[c,d]pyrene	0.000 ± 0.001	<LOD	<LOD			<LOD	0.624		
*chrysene	<LOD	0.001	0.343	0.007	8.727	0.004	2.598	<LOD	3.072
5-methylchrysene	<LOD	0.001	0.822			<LOD	0.055		
*benzo[b]fluoranthene	0.000 ± 0.004	0.001	0.261			<LOD	0.775	<LOD	1.185
*benzo[k]fluoranthene	<LOD	0.001	0.072			<LOD	0.252	<LOD	0.575
benzo[e]pyrene	<LOD	0.003	0.977	<LOD	1.745				
*benzo[a]pyrene	0.015 ± 0.018	0.001	0.333	<LOD	4.655	<LOD	1.009	<LOD	1.229
perylene	0.011 ± 0.020			<LOD	0.604				
*indeno[1,2,3-cd]pyrene	<LOD	0.000	0.156	<LOD	1.455	<LOD	0.359	<LOD	0.589
*dibenz[a,h]anthracene	0.003 ± 0.002	0.000	0.028	<LOD	0.160	<LOD	0.077		
benzo[g,h,i]perylene	0.064 ± 0.263	0.002	0.201	<LOD	1.091			<LOD	0.315
dibenzo[a,l]pyrene	<LOD	0.000	0.000			<LOD	0.015		
dibenzo[a,e]pyrene	0.510 ± 0.656	0.00	0.067			<LOD	0.032		
dibenzo[a,i]pyrene	0.200 ± 0.183					<LOD	0.153		
dibenzo[a,h]pyrene	0.336 ± 0.391					<LOD	0.008		
total PAHs (ng/puff)	1.373	0.120	14.053	0.397	107.164	0.024	78.687	0.374	191.430
BaPeq conc. (pg/puff)	131	9	211	2	944	0	335	0	308

a Benzo­[a]­pyrene-equivalent (BaPeq) concentrations are provided to assess relative carcinogenic potency. Values below the limit of detection are indicated as <LOD. Asterisks (*) denote PAHs classified as Group B2 probable human carcinogens by the U.S. EPA.

b Note: The puff volume is converted to 40 mL for all studies. PAH concentrations in other studies are reported as averages across all EC/TC products.

In addition to the EPA B2 PAHs, our analysis identified dibenzo­[a,e]­pyrene, dibenzo­[a,h]­pyrene, and dibenzo­[a,i]­pyrene as the most abundant PAHs in EC aerosols. Although these dibenzopyrene isomers were not analyzed in most previous EC studies, they possess high toxic equivalency factors (TEFs, Table S2) and thus contribute substantially to overall carcinogenic potential. To quantify cumulative toxicity, we recalibrated concentrations to benzo­(a)­pyrene-equivalent (BaPeq) using TEFs outlined in Table S2, following deposition adjustment as described in section 3.1.1. The BaPeq for ECs was significantly lower than for TCs (Table 4), consistent with the lower B2 PAH concentrations observed. However, the detection of high-TEF dibenzopyrene isomers in EC aerosols underscores a potential health risk that warrants continued attention.

Notably, our recorded total PAH concentration (1.37 ng/puff) was higher than those reported in other EC studies. This discrepancy can be attributed to two factors. First, our more extensive analytical panel included previously unanalyzed dibenzopyrene isomers. Second, the higher operating power of modern EC devices elevates coil temperature, amplifying e-liquid thermal decomposition.^52^ Indeed, Dusautoira, Zarconea^23^ demonstrated that EC devices operating at higher powers produce more PAHs. These findings highlight that reporting the full target list, TEFs, and analytical sensitivity is essential for fair cross-study comparison and accurate risk characterization.

### 3.2 Health Risk Assessment

#### 3.2.1 Excess Lifetime Cancer Risk (ELCR) and Oxidative Potential (OP)

To evaluate the health implications of EC aerosol exposure, ELCR was calculated based on metal concentrations (Table S6) and BaPeq levels (Table 4) for both EC and TC users (Table 5). The ambiguity surrounding the oxidation state of Cr and its notably elevated Slope Factor (SF) necessitates two separate ELCR calculations for EC users. One calculation excludes Cr concentration, while the other includes it assuming hexavalent form. This approach provides a scenario spectrum driven by chromium speciation rather than a single risk estimate. When Cr is excluded, the ELCR value for EC aerosols is approximately 10^–6^, with PAHs emerging as the predominant contributor followed by Ni. However, if Cr levels are incorporated assuming hexavalent form, the ELCR value drastically rises to 10^–3^. These calculations indicate significant cancer risk associated with chronic EC use, particularly under the assumption that chromium exists in its hexavalent form. Given variations in vaping behavior, EC users tend to exhibit distinct puffing topographies compared to TC smokers, including differences in puff duration, puff volume, interpuff interval, and total daily puff frequency.^54^ These behavioral differences can substantially influence delivered dose independent of emission concentration, meaning that risk estimates may differ considerably among occasional and habitual users. Without careful normalization to actual user puffing behavior, comparisons of per-puff emission data between EC and TC may inadvertently under- or overestimate relative exposure burdens. Additionally, the impact of inhaled PAHs on susceptible populations, including adolescents and individuals with pre-existing respiratory conditions, warrants further investigation. To contextualize our findings, Table S7 contrasts our results with prior studies on metal contributions to ELCR in EC aerosols. For perspective, the U.S. EPA^55^ established that the acceptable risk threshold lies between 10^–6^ and 10^–4^, underscoring the critical role of Cr oxidation state in EC aerosol health risk assessments. For TC aerosols, the ELCR is approximately 10^–4^, with Cd as the primary contributor followed by As. Reinforcing this, Stabile, Buonanno^36^ reported that the ELCR for TC smokers is 2–6 × 10^–1^, predominantly influenced by nitrosamines (77–96%). In their analysis, Cd, As, and PAHs (BaPeq) account for merely 12–17%, 2–4%, and 1–2% of the ELCR value, respectively. Our study did not conduct an exhaustive examination of organic compounds in both TC and EC aerosols, which represents a limitation in comprehensive risk characterization.

**Table 5:** ELCR Values and Pollutant Contributions in EC and TC Aerosols^a^

	As	Be	Cr ^6+^	Cd	Co	Ni	Pb	PAHs (BaPeq)	ELCR
EC (this study)	0.0%	0.0%	-	0.0%	0.0%	27.0%	0.2%	72.8%	7.32 × 10^–6^
	0.0%	0.0%	99.8%	0.0%	0.0%	0.1%	0.0%	0.1%	4.15 × 10^–3^
TC	21.3%	0.0%	0.0%	67.0%	0.3%	0.1%	0.1%	11.2%	7.45 × 10^–4^

a Two scenarios for EC account for chromium oxidation state uncertainty (upper row excludes Cr; lower row assumes Cr^6+^). “-” indicates that Cr was excluded from the ELCR calculation.

The oxidative potential (OP) of EC aerosols was assessed to further evaluate their toxicological properties. The DTT assay quantifies the capacity of aerosol constituents to generate reactive oxygen species (ROS). Elevated OP indicates enhanced potential to induce oxidative stress, which is a known contributor to inflammatory responses, endothelial dysfunction, and the progression of respiratory and cardiovascular diseases.^56^ Our analysis revealed an OPv of 49.87 nmol/min/m^3^ and an OPm of 0.02 pmol/min/μg for EC aerosols. Compared to typical atmospheric aerosols, which exhibit OPv ranging from 10^–1^ to 10 nmol/min/m^3^ and OPm ranging from 10 to 10^2^ pmol/min/μg,^33^ the OPv of EC aerosols significantly exceeds ambient levels while the OPm remains notably low. This divergence can be attributed to the physical and chemical characteristics of EC aerosols. EC aerosols are dominated by ultrafine particles with high number concentrations but relatively low mass concentrations, resulting in a large total surface area available for oxidative reactions when normalized to air volume. These findings suggest that particle number and surface area, rather than particle mass alone, are key determinants of EC aerosol toxicity.

#### 3.2.2 Environmental Implications

The health risk assessment presented above reveals several critical considerations for evaluating EC aerosol toxicity. The inclusion of dibenzopyrenes in PAH analysis substantially influences carcinogenic risk estimates. Dibenzopyrenes are recognized as highly carcinogenic PAHs, with TEF values ranging from 1 to 100 depending on the specific isomer.^57^ Previous studies have demonstrated that dibenzopyrenes contribute substantially to the carcinogenic potency of PAH mixtures, and that risk assessments excluding these compounds may significantly underestimate total cancer risk.^58^ Despite their toxicological importance, dibenzopyrenes are seldom included in routine PAH monitoring due to analytical challenges associated with their low concentrations and high molecular weight.^59^ Our results demonstrate that BaPeq values increase substantially when dibenzopyrenes are included, confirming that analytical panel scope explains much of the cross-study variance even when total PAH concentrations appear similar.

Cr speciation emerges as the most consequential uncertainty in EC health risk assessment. ELCR estimates span 3 orders of magnitude (10^–6^ to 10^–3^) depending on whether chromium exists as Cr^3+^ or Cr^6+^, representing the difference between negligible and unacceptable risk under U.S. EPA guidelines. A key limitation of this study is the absence of empirical Cr speciation data. The ICP-MS method employed quantifies total Cr concentration but cannot distinguish between Cr^3+^ and Cr^6+^, which differ substantially in carcinogenic potency. Future studies should incorporate speciation-specific techniques such as X-ray absorption near-edge structure (XANES) spectroscopy to determine the actual Cr^6+^ fraction in EC aerosols and provide more definitive ELCR estimates. Additionally, the PSDs measured under ambient conditions were used as MPPD input without accounting for hygroscopic growth within the respiratory tract, which likely causes our deposition fraction and corresponding ELCR estimates to represent conservative lower bounds.

The divergence between OPv and OPm further highlights that particle number and surface area, rather than mass alone, are key determinants of EC aerosol toxicity. Dual reporting of both metrics is therefore essential to avoid normalization artifacts and to accurately characterize oxidative stress. It is also important to acknowledge that the carcinogenic risk estimates presented here likely represent a conservative lower bound. Alkylated, oxygenated, and nitrated PAH derivatives were not quantified and may contribute to cumulative carcinogenic burden, while TEF-based extrapolations for several detected high-molecular-weight congeners may not fully capture their biological activity. Moreover, the present framework does not account for potential mixture interactions among EC aerosol constituents, and emerging evidence suggests that coexposures to PAHs, metals, and carbonyls may produce additive or synergistic effects not captured in single-compound models, implying that overall health risks may be systematically underestimated.

In summary, these findings demonstrate that EC health risks are device-dependent and end point-specific. Accurate risk characterization requires comprehensive analytical panels, speciation-specific measurements, standardized reporting protocols, and future frameworks capable of capturing mixture-level toxicity.

## 4 Conclusions

This study provides a comprehensive assessment of EC aerosol health risks by integrating chemical characterization, ELCR, and OP measurement. Metal analysis identified five metals (Cu, Ni, Ag, Zn, Pb) exclusively in aerosols, indicating device components as primary contamination sources. Cross-study comparison revealed substantial variability in metal profiles arising from manufacturing differences and device design, highlighting the device-dependent nature of EC metal exposure. The influence of analytical methodology on risk estimates was further demonstrated through PAH analysis. Inclusion of high-TEF dibenzopyrenes substantially elevated BaPeq-based risk estimates, underscoring the critical role of analytical panel selection in carcinogenic risk assessment. Similarly, ELCR calculations revealed that Cr speciation represents the dominant source of uncertainty, with estimates spanning 3 orders of magnitude (10^–6^ to 10^–3^) depending on the assumed oxidation state. OP analysis demonstrated marked divergence between OPv and OPm, suggesting that particle number and surface area, rather than mass alone, drive EC aerosol toxicity. These findings demonstrate that EC health risks are device-dependent and end point-specific, requiring assessment approaches distinct from those applied to TC emissions. Future research should prioritize standardized analytical protocols, speciation-specific measurements, and harmonized reporting practices to enable accurate risk characterization.

## Supplementary Material



## Notes

Detailed information on the experimental setup for EC aerosol generation; method detection limits for 19 target elements in e-liquid and aerosol samples; toxic equivalence factors and detection limits of PAHs; slope factors of carcinogenic compounds; metal levels in EC aerosols and e-liquid; metal concentrations per puff in EC aerosol and a comparison with traditional cigarettes; and excess lifetime cancer risk values contributed by metals in EC aerosols (PDF)
