# Supplementary material for: Comprehensive Health Risk Assessment of Electronic Cigarette Aerosols: Metal and PAH Characterization, Oxidative Potential, and Cancer Risk Estimation
Source: ACS Environ Au. 2026 Apr 15;6(4):634–42. doi: 10.1021/acsenvironau.5c00304 (PMC13377506; doi:10.1021/acsenvironau.5c00304)
Supplement: Supplementary file 1 [file vg5c00304_si_001.pdf]

Supporting Information

Comprehensive Health Risk Assessment of Electronic Cigarette Aerosols: Metal and PAH Characterization, Oxidative Potential, and Cancer Risk Estimation

Li-Ti Chou[1], Tsai-Ling Chen[1], Jen-Kun Chen[2], Hsiao-Chi Chuang[3,4,5,6], Kai-Chien Yang[7,8,9,10] and Ta-Chih Hsiao[1,11,12*]

[1]Graduate Institute of Environmental Engineering, National Taiwan University, Taipei,106319, Taiwan

[2]Institute of Biomedical Engineering and Nanomedicine, National Health Research Institutes, Miaoli, 350401, Taiwan

[3]School of Respiratory Therapy, College of Medicine, Taipei Medical University, Taipei, 110301, Taiwan

[4]National Heart and Lung Institute, Imperial College London, London, SW3 6LY, United Kingdom

[5]Division of Pulmonary Medicine, Department of Internal Medicine, Shuang Ho Hospital, Taipei Medical University, New Taipei City, 235041, Taiwan

[6]Cell Physiology and Molecular Image Research Center, Wan Fang Hospital, Taipei Medical University, Taipei, 110301, Taiwan

[7]Department and Graduate Institute of Pharmacology, National Taiwan University College of Medicine, Taipei, 106319, Taiwan

[8]Research Center for Developmental Biology & Regenerative Medicine, National Taiwan University, Taipei, 106319, Taiwan

[9]Division of Cardiology, Department of Internal Medicine and Cardiovascular Center, National Taiwan University Hospital, Taipei, 100225, Taiwan

[10]Institute of Biomedical Sciences, Academia Sinica, Taipei, 115201, Taiwan

[11]Research Centre for Environmental Changes, Academia Sinica, Taipei, 115201, Taiwan

[12]Institute of Atomic and Molecular Sciences, Academia Sinica, Taipei, 115201, Taiwan

[*]Corresponding Author: Ta-Chih (T.C.) Hsiao, tchsiao@ntu.edu.tw

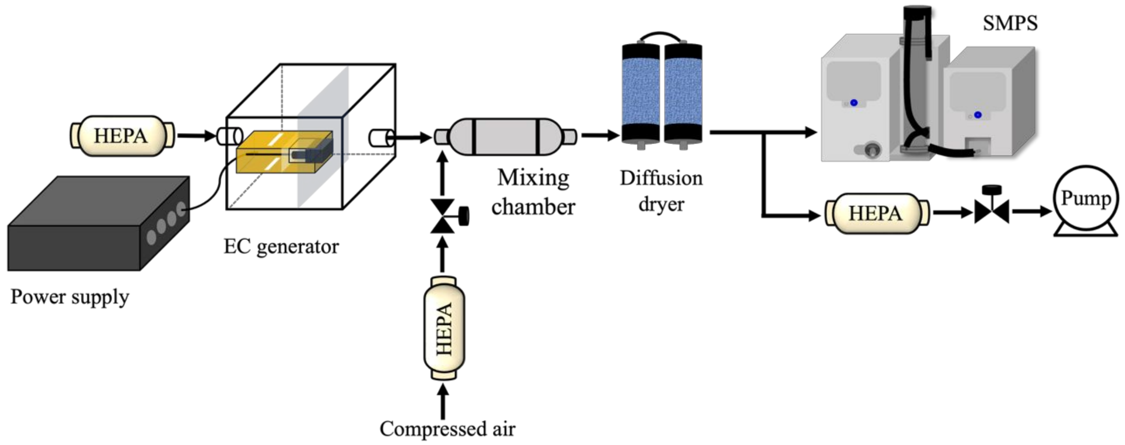

Figure S1. Schematic of the experimental setup for EC aerosol generation.

Table S1. Method detection limits (MDLs) for 19 target elements in e-liquid and aerosol samples.

	Al	Ba	As	Cr	Cd	Cs	Co	Cu	Ga	In	Mn	Ni	Se	Ag	Tl	V	Zn	Pb	Fe
E-liquid (μg/kg)	0.58	0.05	0.15	0.09	0.001	0.001	0.003	0.44	0.02	0.004	0.03	0.41	0.004	0.03	0.02	0.01	0.33	0.10	1.61
Aerosol (μg/kg)	0.89	0.13	0.02	0.52	0.001	0.001	0.01	0.60	0.003	0.001	0.15	0.04	0.01	0.03	0.001	0.02	0.78	0.09	9.63

Table S2. Toxic equivalence factor (TEFs) and detection limit of PAHs.

PAH compound	TEF*	DL (pg)	PAH compound	TEF*	DL (pg)
Naphthalene	0.001	0.093	5-Methylchrysene	1	
2-Methylnaphthalene	0.001		Benzo[b]fluoranthene	0.1	0.030
Acenaphthylene	0.001	0.016	Benzo[k]fluoranthene	0.1	0.033
Acenaphthene	0.001		Benzo[e]pyrene	0.01	
Fluorene	0.001		Benzo[a]pyrene	1	0.048
Phenanthrene	0.001	0.052	Perylene	0.001	
Anthracene	0.01	0.060	Indeno[1,2,3-cd]pyrene	0.1	0.084
Fluoranthene	0.001	0.030	Dibenz[a,h]anthracene	1	0.050
Pyrene	0.001		Benzo[g,h,i]perylene	0.01	0.091
Benzo[c]fluorene	20		Dibenzo[a,l]pyrene	30	
Benz[a]anthracene	0.1	0.012	Dibenzo[a,e]pyrene	0.4	
Cyclopenta[c,d]pyrene	0.1		Dibenzo[a,i]pyrene	0.6	
Chrysene	0.01	0.013	Dibenzo[a,h]pyrene	0.9	

*TEF value is referred to Yang [1].

Table S3. The slope factors (SFs) of carcinogenic compound.

Chemical	As	Be	Cr^{6+}	Cd	Co	Ni	Pb	PAHs (BaPeq)
SF $(mg/kg\text{-}day)^{-1}$	12	8.4	510	15	27	0.91	0.042	3.9

Table S4. Metal levels in EC aerosols and e-liquid (µg/kg).

	Al	Ba	As	Cr	Cd	Cs	Co	Cu	Ga	In	Mn	Ni	Se	Ag	Tl	V	Zn	Pb	Fe
E-liquid	0.64	0.07	<LOD	0.48	<LOD	<LOD	<LOD	<LOD	<LOD	<LOD	0.01	<LOD	0.002	<LOD	<LOD	0.01	<LOD	<LOD	0.37
Mean (SD)	(0.29)	(0.003)	(<0.15)	(0.31)	(<0.001)	(<0.001)	(<0.003)	(<0.44)	(<0.02)	(<0.004)	(0.003)	(<0.41)	(0.002)	(<0.03)	(<0.02)	(0.002)	(<0.33)	(<0.10)	(0.25)
> LOD ratio (n = 5)	80%	40%	0%	100%	0%	0%	0%	40%	0%	0%	60%	100%	80%	40%	0%	100%	80%	80%	60%
Aerosol	0.89	0.03	<LOD	0.57	<LOD	<LOD	<LOD	0.03	<LOD	<LOD	0.06	0.15	0.01	0.20	<LOD	0.01	0.22	0.03	2.37
Mean (SD)	(0.66)	(0.01)	(<0.02)	(0.17)	(<0.001)	(<0.001)	(<0.01)	(0.03)	(<0.005)	(<0.001)	(0.06)	(0.05)	(0.01)	(0.26)	(<0.001)	(0.01)	(0.06)	(0.04)	(2.15)

Note: The average concentrations of each metal in the aerosol were calculated using only the quantifiable values (no substitutions were made for value <LOD). LOD value is the blank sample concentration.

Table S5. Metal mean (standard deviation) levels in EC aerosol (ng/puff).

	Al	Ba	As	Cr	Cd	Cs	Co	Cu	Ga	Mn	Ni	Se	Ag	Tl	V	Zn	Pb	Fe	Sb	Sn
This study[a] (n = 5)	6.57 (4.85)	0.24 (0.10)	<LOD (<0.17)	4.20 (1.23)	<LOD (<0.002)	<LOD (<0.01)	<LOD (<0.09)	0.23 (0.22)	<LOD (0.03)	0.41 (0.43)	1.12 (0.36)	0.06 (0.05)	1.49 (1.91)	<LOD (<0.01)	0.06 (0.04)	1.64 (0.42)	0.23 (0.32)	17.38 (15.81)	—	—
Beauval, Antherieu [2] (n = 18)	—	—	< LOQ (<0.01)	0.09	0.002	—	—	—	—	—	—	—	—	—	—	—	0.04	—	0.01	—
Palazzolo, Crow [3] (n = 8)	345.71	—	0.16	—	< LOD	—	—	< LOD	—	< LOD	17.22	—	—	—	—	73.73	< LOD	0.08	—	—
Olmedo, Goessler [4] (n = 56)	0.01 (0.03)	—	0.002 (0.01)	0.04 (0.16)	0.0001 (0.002)	—	—	0.03 (0.07)	—	0.01 (0.01)	0.19 (0.80)	—	—	—	—	0.32 (0.53)	0.05 (0.16)	0.23 (0.80)	0.001 (0.002)	0.01 (0.04)

Note: Puff volume for all studies has converted into 40 mL. —, not measured; LOD, the limit of detection; LOQ, limit of quantification. The data for Beauval, Antherieu [2], Palazzolo, Crow [3], and Olmedo, Goessler [4] are obtained from Zhao, Aravindakshan [5]. a: LOD value in this study is the blank sample value.

Table S6. Metal mean (standard deviation) levels in EC and TC (ng/puff).

	Al	Ba	As	Cr	Cd	Cs	Co	Cu	Ga	Mn	Ni	Se	Ag	Tl	V	Zn	Pb	Fe
This study	6.57 (4.85)	0.24 (0.10)	<LOD (<0.17)	4.20 (1.23)	<LOD (<0.002)	<LOD (<0.01)	<LOD (<0.09)	0.23 (0.22)	<LOD (0.03)	0.41 (0.43)	1.12 (0.36)	0.06 (0.05)	1.49 (1.91)	<LOD (<0.01)	0.06 (0.04)	1.64 (0.42)	0.23 (0.32)	17.38 (15.81)
Palazzolo, Crow [3]	—	—	0.17 (0.01)	—	1.80 (0.08)	—	—	—	—	—	—	—	—	0.04 (0.01)	—	—	0.66 (0.02)	—
Beauval, Antherieu [2]	634.92	—	4.76	—	6.35	—	—	3.97	—	158.73	—	—	—	0.00	—	79.37	0.79	0.00
Pappas, Fresquez [6][a]	—	—	0.46 (0.04)	<LOD (0.13)	5.39 (0.38)	—	0.01 (0.001)	—	—	0.18 (0.02)	0.07 (0.01)	—	—	—	—	—	2.02 (0.13)	—

Note: Puff volume for all studies has converted into 40 mL. —, not measured; LOD, the limit of detection. a: Only calculated the ISO smoke regimen results and assumed that eight puffs were produced per cigarette.

Table S7. ELCR value of EC aerosols contributed by metals.

	This study	Beauval, Antherieu [2]	Palazzolo, Crow [3]	Olmedo, Goessler [4]
Exclude Cr	1.99×10^{-6}	4.93×10^{-8}	3.40×10^{-5}	3.99×10^{-7}
Include Cr	4.14×10^{-3}	9.25×10^{-5}	3.40×10^{-5}	4.18×10^{-5}

Reference

1. Yang, H.Y., Hazardous Air Pollutants in Fine Particulate Matters：Source Apportionment and Exposure Risk Assessment at Different Areas in Taiwan, in Master Thesis. 2018, National Yang-Ming University.
2. Beauval, N., et al., Chemical evaluation of electronic cigarettes: multicomponent analysis of liquid refills and their corresponding aerosols. Journal of analytical toxicology, 2017. 41(8): p. 670-678.
3. Palazzolo, D.L., et al., Trace metals derived from electronic cigarette (ECIG) generated aerosol: potential problem of ECIG devices that contain nickel. Frontiers in physiology, 2017. 7: p. 663.
4. Olmedo, P., et al., Metal concentrations in e-cigarette liquid and aerosol samples: the contribution of metallic coils. Environmental health perspectives, 2018. 126(2): p. 027010.
5. Zhao, D., et al., Metal/metalloid levels in electronic cigarette liquids, aerosols, and human biosamples: a systematic review. Environmental health perspectives, 2020. 128(3): p. 036001.
6. Pappas, R.S., et al., Toxic metal concentrations in mainstream smoke from cigarettes available in the USA. Journal of analytical toxicology, 2014. 38(4): p. 204-211.
